# A Report of a Rare Case of BCOR (B-cell Line 6 Corepressor)-Altered Sarcoma of the Lung in a Child

**DOI:** 10.7759/cureus.59731

**Published:** 2024-05-06

**Authors:** Jasmin Capellan, Sruti Pari, Lalita Ganti, Charles Li, Sathyaprasad Burjonrappa

**Affiliations:** 1 Pediatric Surgery, Rutgers Robert Wood Johnson Medical School, New Brunswick, USA; 2 Pediatrics, Rutgers Robert Wood Johnson Medical School, New Brunswick, USA

**Keywords:** r0 resection, paediatric chest wall resection, mediastinal mass, bcor-altered sarcoma, ewing’s sarcoma

## Abstract

Ewing sarcoma is one of the small round blue cell tumors of childhood that typically affects bone. Recently, a subgroup of undifferentiated round-cell sarcomas has been genetically identified as BCOR (B-cell Line 6 Corepressor)-altered sarcomas (BAS). We present a case of a six-year-old male child who presented with a chief complaint of shortness of breath and tachypnea and was found to have a large mediastinal mass concerning sarcoma. Preliminary biopsy results were positive for small round blue cells, possibly Ewing sarcoma. After six cycles of chemotherapy, with subsequent shrinkage of mediastinal mass, the patient was able to undergo wedge resection and excision of the mass with en bloc resection of the fifth and sixth rib, preserving his right lung. Final tissue pathology was positive for BAS. There have been only four reported cases of BAS of the chest wall and zero reported cases of primary tumor presentation of the lung, making this a rare presentation of the disease.

## Introduction

Ewing sarcoma (ES) is the second-most common primary malignant bone tumor in the pediatric population, following osteosarcoma, with the most frequently affected population being aged 10-19 years and of Caucasian descent [1]. ES comprises a spectrum of neoplasms, sometimes referred to as the “Ewing Family of Tumors” due to the shared histopathological features of the malignancies that point towards a common mesenchymal stem cell [2]. More recently the WHO updated small round cell tumor classifications, identifying BCOR (B-cell Line 6 Corepressor)-alerted and CIC (Capicua) rearranged sarcomas as their own entities [3]. Most BCOR-altered sarcomas (BAS) have skeletal origins, though other sites are rarely affected including the lung [3]. Tumors of the ES family are characterized by non-random gene translocations that result in the production of overactive transcription factors [4]. Patients with ES tumors present with pain, swelling, and/or stiffness in the affected area for weeks to months. The various locations of the tumor present with different symptoms: pathological fractures due to bone lesions, back pain referred from the pelvis, asymmetric lung sounds or rales due to lung and pleura involvement, or thrombocytopenia due to bone marrow metastases [5]. Treatment and management of BCOR-sarcomas are currently based on those used for ES. ES has been the subject of many recent clinical trials, with research leading to alternating vincristine-doxorubicin-cyclophosphamide and ifosfamide-etoposide (VDC/IE) cycles every two weeks with local tumor treatment of surgery resection and/or radiation becoming standard of care [6]. Tumors in distal extremities fare better than those more proximal, while solitary pulmonary metastases have a better prognosis than extrapulmonary metastatic sites. Positive prognostic factors include diagnosis before age 15 and minimal to no viable residual tumor following surgical resection [7,8]. Current evidence suggests that the prognosis of BAS, with survival rates of 72-80% after five years, is similar to that of ES [9]. Here, we present a rare case of BAS originating in the lung tissue of a six-year-old boy.

## Case presentation

**Figure 1:**
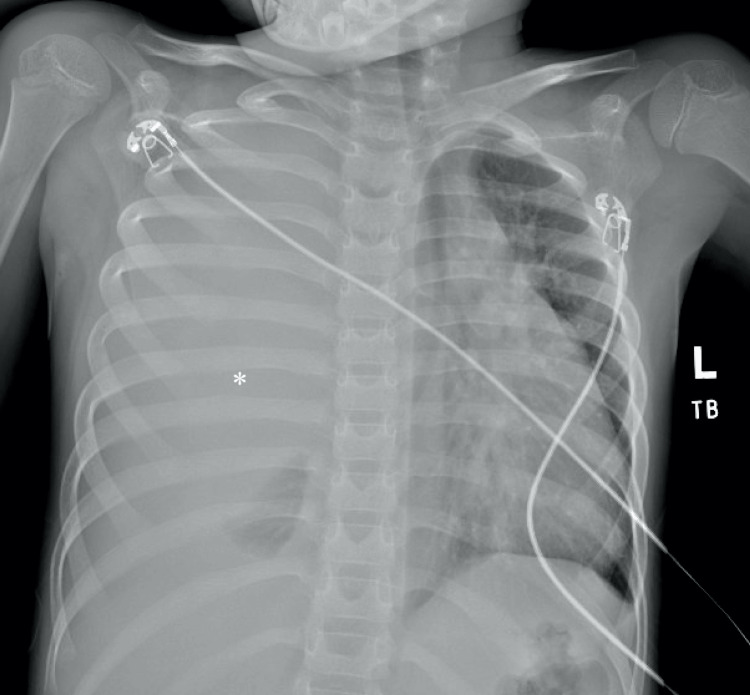
CXR showing opacity in the right hemithorax Large right-sided chest mass with mediastinal shift

A six-year-old boy initially presented to an outside hospital (OSH) for a three-week history of progressive shortness of breath and tachypnea. Prior to transfer, an X-ray was concerning for a right hemithorax, and the patient was transferred. The patient was noted to be tachycardic, tachypneic, saturating in the low 90s, and febrile at 104°F. Labs were significant for elevated lactate dehydrogenase (LDH) 1502 and marginally elevated calcium. Prior to admission, the patient had decreased oral intake with two episodes of non-bilious emesis. He had no sick contacts and was up to date on all vaccinations. On review of systems, the patient’s caregiver reported cough, increased work of breathing, and congestion. There was no past surgical history and no family history of cancer. Repeat chest X-ray demonstrated near complete opacification of the right hemithorax with mediastinal shift to the left (Figure 1).

Upon transfer to our emergency department, the physical exam was positive for diminished breath sounds on the right upper and middle lobes with coarse breath sounds on the left. Follow-up imaging with a chest computed tomography (CT) revealed a large mass in the right hemithorax with invasion of the right sixth rib and chest wall, pleural metastases, and loculated malignant pleural effusion. On admission to the pediatric intensive care unit for management of the mediastinal mass with biopsy and bone marrow aspirate, the patient remained tachycardic in the 130s and tachypneic in the 40s with saturations in the 90s. His increased work of breathing improved after being placed on 2L/min of oxygen on a nasal cannula.

**Figure 2:**
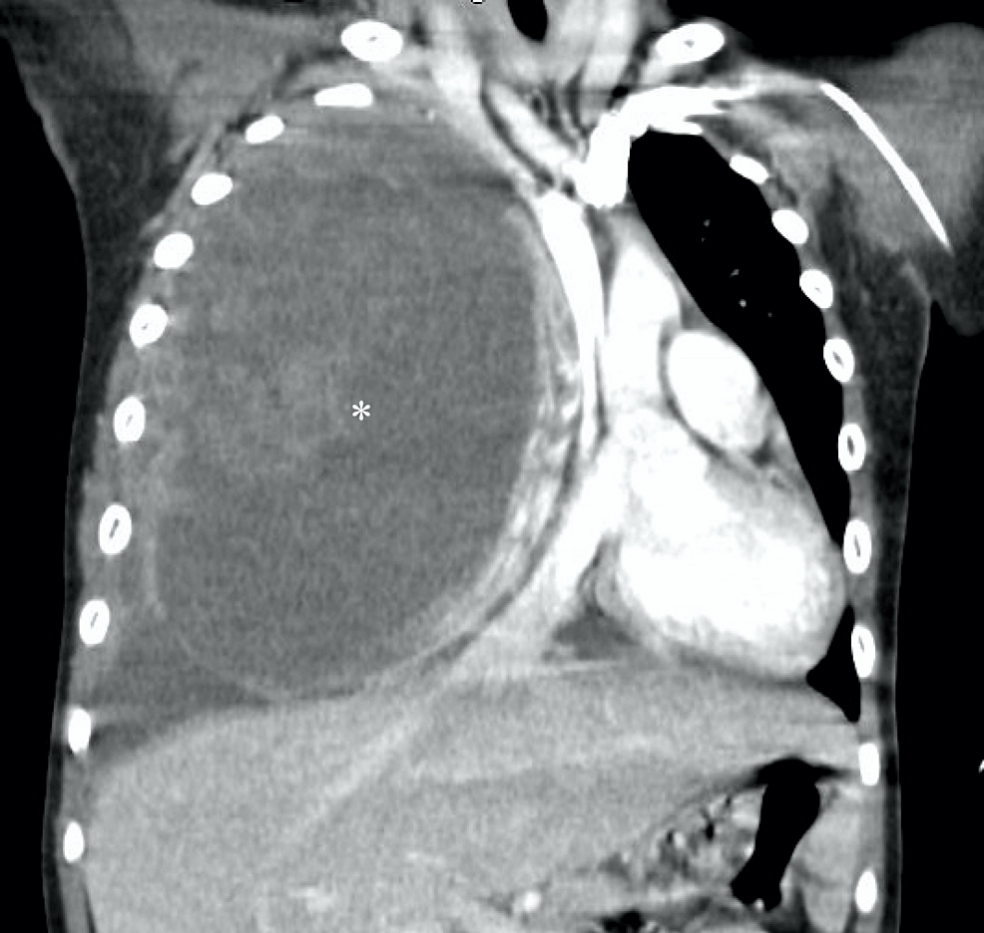
CT scan showing a large mass occupying almost the entire right thoracic cavity with mediastinal displacement Mass measures approximately 13 cm AP by 10 cm transverse by 15 cm craniocaudal. There is evidence of invasion of the lateral right sixth rib and chest wall. There is a near-complete collapse of the right lung with a mediastinal shift to the left.

On review of the imaging (Figures 1-2), the mass was concerning for malignancy, including ES, rhabdomyosarcoma, teratoma, and lymphoma. His laboratory results are indicated in Table 1. Further workup was planned to understand the extent of disease. Bone scan was negative for metastasis. Preliminary bone marrow results were positive for small round blue cells, possibly ES. Due to the patient’s pulmonary compromise and the size of the tumor, the team decided on chemotherapy as soon as central line access was obtained with parental consent.

**Table 1:** Laboratory parameters in the patient The patient had an elevated LDH and calcium level. The LDH elevation suggested a malignant process while the elevated calcium levels suggested metastatic disease. * indicates values that were outside of the laboratory reference range. The Calcium, LDH, and Sodium were abnormal secondary to the tumor. The creatinine was low because the patient was a child. The glucose level was normal on subsequent testing. BUN: blood urea nitrogen; LDH: lactate dehydrogenase

Lab Value	Result	Lab Reference
Sodium	131* mmol/L	136-145 mmol/L
Potassium	4.3 mmol/L	3.5-5.1 mmol/L
Chloride	102 mmol/L	98-110 mmol/L
Bicarbonate	26 mmol/L	21.0-32.0 mmol/L
BUN	7 mg/dL	6.0-18.0 mg/dL
Creatinine	0.36* mg/dL	0.700-1.300 mg/dL
Glucose	119* mg/dL	74-106 mg/dL
Calcium	11.8* mg/dL	8.5-10.1 mg/dL
Uric Acid	4.3	1.2-5.1 mg/dL
LDH	1502*	87-241 Unit/L
WBC	12.8	5.0-13.0 Thousand/mcL
Hemoglobin	9.9	12.5-14.2 g/dL
Hematocrit	29.7	36.0-47.0%
Platelets	425	150-450 Thousand/mcL

**Figure 3:**
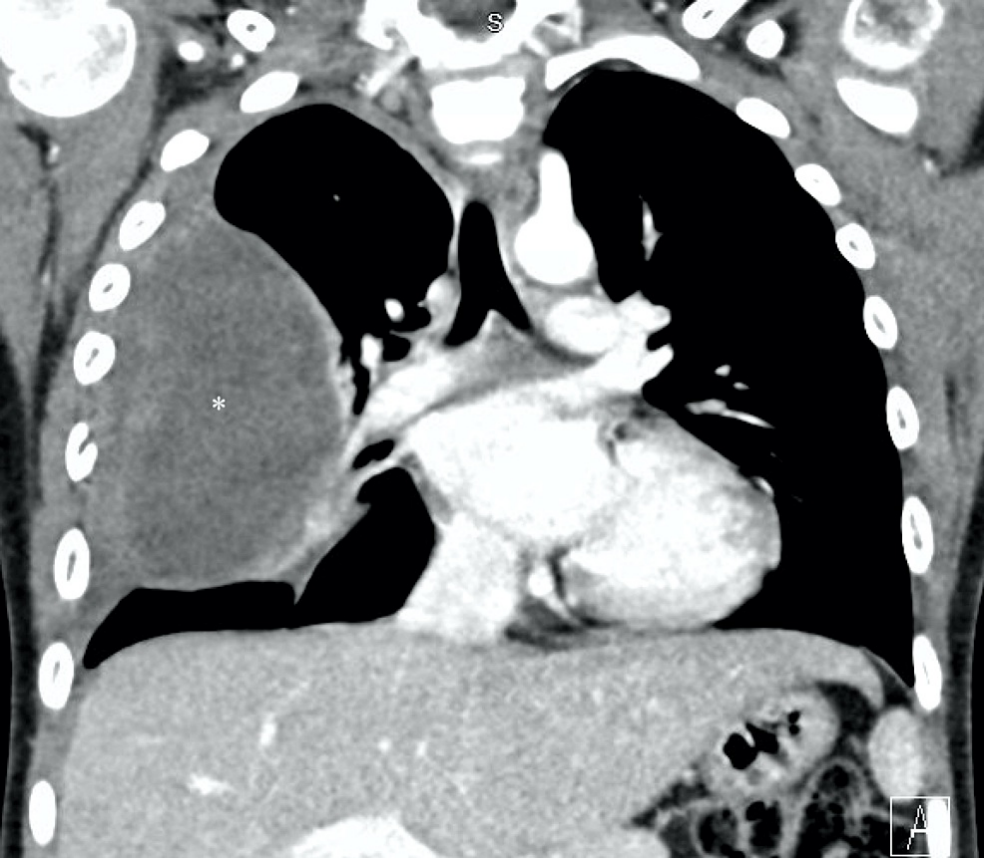
CT scan showing improved aeration of the right lung after six cycles of chemotherapy and shrinkage of the mass Mass now measuring 9.4x5.6x8.8 cm.

The patient was placed on the Adolescent Ewing Sarcoma Working Group (AEWS) 1031 treatment protocol, a phase III randomized trial of adding vincristine-topotecan-cyclophosphamide to standard chemotherapy in the initial treatment of ES. Standard chemotherapy included ifosfamide/etoposide and vincristine/doxorubicin/Cytoxan. Induction chemotherapy was started with doxorubicin, Cytoxan, Zinecard, Mensa and vincristine followed by Zinecard and doxorubicin. Zinecard was used to decrease the cardiotoxicity effect of doxorubicin. The dosing interval was compressed based on the prior AEWS0032 study for better efficacy. This hospitalization for induction of his chemotherapy regimen was complicated by bacteremia, respiratory distress, fever, rhinovirus, and thrombus in the left upper extremity. The patient recovered and a repeat chest CT after a total of six cycles of chemotherapy revealed a shrinking mass (Figure 3). The final biopsy results were positive for an atypical small round cell mediastinal mass. Pediatric surgery was consulted for further management and consent was obtained for a thoracotomy and resection of the mass and possible total right pneumonectomy due to involvement of hilar structures.

**Figure 4:**
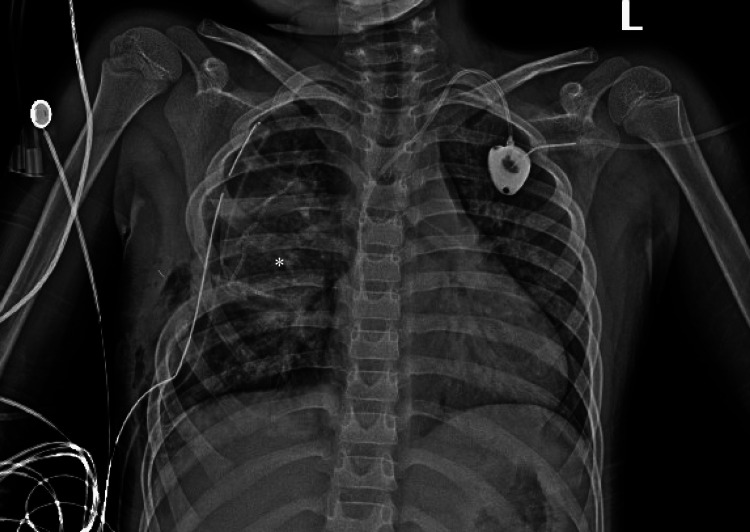
CXR showing the expansion of the right lung after resection of mass en bloc with the fifth and sixth ribs.

Intraoperatively, the patient was placed in the left lateral decubitus position with appropriate padding of bony prominences. Anesthesia attempted isolated left lung intubation, but this was not successful. We started with an extrapleural pneumonectomy approach due to the involvement of the pleura and ribs. The fifth and sixth ribs were partially resected, where they were infiltrated by the tumor, en bloc with the specimen. As we started working on the inferior pulmonary ligament, we noticed that every time the middle and lower lobes were compressed to perform the dissection, the patient was desaturated. We recognized that the patient would not tolerate a pneumonectomy. We opened the mediastinal pleura and examined the hilar structures and noted that the tumor mainly involved the pleura, chest wall, and upper lobe and did not invade the hilar structures. Further, once the pleural mass was separated from the middle and lower lobes and the major fissure was dissected and completed with a stapling device most of the pulmonary parenchyma could be preserved. A wedge resection of the right upper lobe allowed for complete resection of the mass with subtotal pleural resection. Cryoablation at five intercostal levels was administered for pain control. Post-operative chest X-ray revealed good expansion of the lung (Figure 4).

Tissue pathology confirmed mesenchymal sarcoma most compatible with BAS. Consolidation chemotherapy per the AWES 1031 protocol which included radiation therapy in this case is ongoing. The patient has not had any evidence of recurrence at his four-month follow-up.

## Discussion

Approximately 200 children and teens are diagnosed with a tumor in the Ewing family in the United States each year [1]. Even more rare is primary pulmonary ES as described by Fedeli et al. who identified 50 cases of primary pulmonary ES in the last decade. BAS are rare with a much lower incidence than ES [10]. In our literature review, of the total 169 cases of BAS identified, no cases were identified with the primary tumor site of the lung. Only four had a primary site of the chest wall [1,11,12]. Most cases are present in skeletal bone or soft tissue. Of note, these tumors predominantly occur in males with a ratio of 3.4:1 compared to a ratio of 1.4:1 for ES [11]. Studies have also shown a statistically significant nine-fold difference in the incidence of ES between Caucasians and African Americans [1]. Our case of a six-year-old African-American boy with a right-sided mediastinal mass is a rare presentation of the disease both in tumor location and pathology. A summary of all the identified cases is provided in Table 2.

**Table 2:** Summary of reported cases of pediatric BCOR-altered sarcomas *adult cases were excluded from this summary; **ITD: internal tandem duplication of PUFD domain, a BCOR alteration

Author(s), Year	Cases	Sex	Age(s)	Tumor Site	Molecular
Tramontana et al., 2020 [2]	1	M	1	bone	BCOR::CCNB3 fusion
Pierron et al., 2012 [4]	24	16M, 8F	6-26	bone, 1 chest wall	BCOR::CCNB3 fusion
Cohen-Gogo et al., 2014 [7]	26	M	6-26	bone, soft tissue	BCOR::CCNB3 fusion
Peters et al., 2015 [11]	6	M	7-13	bone, 1 chest wall	BCOR::CCNB3 fusion
Puls et al., 2014 [12]	10	9M, 1F	11-18	bone, soft tissue	BCOR::CCNB3 fusion
Matsuyama et al., 2017 [13]	11	M	6-18	bone	BCOR::CCNB3 fusion
Kao et al., 2018 [14]	35*	31M, 5F	2-24	bone, 2 chest wall	BCOR::CCNB3 fusion
Salgado et al., 2022 [15]	27	19M, 8F	0-10	bone, soft tissue	BCOR-ITD**
Sarper-Sauer et al., 2023 [16]	29	23M, 6F	0-19	bone, soft tissue	BCOR-ITD**, BCOR::CCNB3 fusion, MAML::BCOR STS fusion

Due to an initial chest X-ray indicating complete opacification of the right hemithorax, our patient underwent a chest CT revealing a large heterogenous lobar mass measuring 13 cm AP by 10 cm transverse by 15 cm craniocaudal with complete collapse of the right lung. Though none of the cases described by Cohen-Gogo et al. originated in the lungs, the soft tissue tumors they identified manifest similar characteristics of heterogeneous masses with intense enhancement on CT [7,17]. Sirisena et al. summarized the imaging in 148 cases of BAS, concluding that the typical imaging of soft tissue masses reveals well-demarcated or ill-defined borders, but all were deep to the fascia or within the muscle belly [17]. Diagnostic imagining in combination with pathology and molecular testing is needed to confirm the diagnosis of BAS. In our patient, we initially considered pleuropulmonary blastoma (PPB) to be excluded after histopathology showed small round blue cells.

BAS were first identified by Pierron et al. in 2012, previously classified as an undifferentiated small round cell sarcoma [4]. Ewing-like sarcomas (ELS) share significant morphology and immunohistochemistry with ES, making differential diagnosis challenging. In our case, the initial biopsy showed small round cells that were suggestive of ES. Currently, BAS are treated with ES regimens and exhibit similar survival rates of 72-80% after five years [9]. Kao et al. identified the morphological similarities between BAS and clear cell sarcoma of the kidney (CCSK), which responds to chemotherapy with less toxicity than ES treatment regimens [14]. Identifying molecular aberrations can help identify potential targets for future novel therapies [5]. However, molecular testing is not standard practice for these tumors as Fedeli et al. found that fluorescence in situ hybridization (FISH) and molecular testing were scantly performed in the 50 cases of primary pulmonary ES cases they identified [10]. Histologically, BAS are identified as primitive round and spindle cells in sheets, nests, and fascicles [15]. In the 36 cases identified by Kao et al., 78% showed small round cells, while the other 22% were of spindle-like morphology on histology. Regardless of morphology, they showed high rates of a rich capillary network [14]. The variability in histologic presentation is distinctive from ES, which is characterized by a solid sheet of monomorphic round cells, making narrowing the differential possible [18]. Nonetheless, overlap with other ELS tumors such as CDS (Capicua:DUX4 fusion oncoprotein positive sarcoma) exists, and identification of BCOR special AT-rich sequence-binding protein 2 (SATB2) and cyclin D1 positivity on immunohistochemistry serves to further narrow the differential [19].

The overall survival rate of ES is 63% although there have been case reports of rapidly progressive disease [20]. Al-Marshad et al. described a case of rapidly progressive metachronous skeletal metastatic ES within four weeks of diagnosis without evidence of pulmonary involvement [20]. Of note, Pierron et al. did find that nine out of 21 cases had metastasis to the lung, with the primary tumor site as skeletal bone [4]. In the 169 BAS cases we identified, the most common site of recurrence was the lung. Despite similar clinical presentations and chemo-responsiveness, ES and BAS seem to behave differently in terms of the progression of the disease. Novel therapies have shown benefits in the treatment of BAS. Tramontana et al. described a pediatric case of BAS that showed benefit with more than two years of disease-free remission and argued for further research using CDK4/6 inhibitors in BAS [2]. Understanding the histological presentation of the disease and identifying alterations in undifferentiated sarcomas on immunohistochemistry and molecular testing serves to help delineate the prognostic significance of BCOR alterations and develop targeted treatments.

## Conclusions

To our knowledge, this is the first report of the lung as the primary tumor site of BAS in the pediatric literature. Further, it highlights the importance of immunohistochemistry and genetic studies to distinguish between different ELS as treatment strategies and responses vary based on the genetic abnormality that underlies tumor biology.
